# Family experiences and attitudes toward care of ICU patients with delirium: A scoping review

**DOI:** 10.3389/fpubh.2022.1060518

**Published:** 2022-11-23

**Authors:** Sandra Lange, Wioletta Mȩdrzycka-Da̧browska, Adriano Friganović, Dorota Religa, Sabina Krupa

**Affiliations:** ^1^Department of Internal and Pediatric Nursing, Medical University of Gdańsk, Gdańsk, Poland; ^2^Department of Anaesthesiology Nursing and Intensive Care, Faculty of Health Sciences, Medical University of Gdańsk, Gdańsk, Poland; ^3^Department of Anesthesiology and Intensive Medicine, University Hospital Centre Zagreb, Zagreb, Croatia; ^4^Department of Nursing, University of Applied Health Sciences, Zagreb, Croatia; ^5^Division for Clinical Geriatrics, Department of Neurobiology, Care Sciences and Society (NVS), Karolinska Institute, Solna, Sweden; ^6^Institute of Health Sciences, College of Medical Sciences of the University of Rzeszow, Rzeszow, Poland

**Keywords:** family, experiences, delirium, ICU, scoping review

## Abstract

**Introduction:**

The family has an important role in the care of the ICU patient. Research shows that the implementation of non-pharmacological interventions to prevent delirium, including interventions with the family, can reduce the incidence of delirium. The aim of this review was to search the available literature about the experiences and attitudes of family/carers of ICU patients diagnosed with delirium during hospitalization.

**Methods:**

A scoping review method was used to map terms relevant to the involvement of relatives in the care of critically ill patients with delirium. To identify studies, the following databases were searched: PubMed, Scopus, EBSCO, Web of Science, and Cochrane Library. The database search was ongoing from 15 July 2022, with a final search on 4 August 2022.

**Results:**

Thirteen articles reporting on the experiences and attitudes of family/carers of ICU patients who developed delirium during hospitalization were included in the scoping review. Of the included studies, eight were qualitative studies, three were quantitative studies and two were reviews (systematic review and integrative review). The studies were conducted in North America, Europe, South Africa, and Asia. Our findings show that carers experienced adverse effects associated with delirium in ICU patients such as stress, anxiety, embarrassment, uncertainty, anger, shock. Families/relatives need both emotional and informational support from medical staff.

**Conclusion:**

Relatives want to be involved in the care of the delirium patient, although this needs improvement in some aspects of care such as: lack of awareness, family/relatives knowledge of delirium, improved education, and communication with medical staff. Recognition of delirium by families is acceptable and feasible. Family involvement may induce an increased anxiety, but this aspect needs further research.

## Introduction

**Figure 1:**
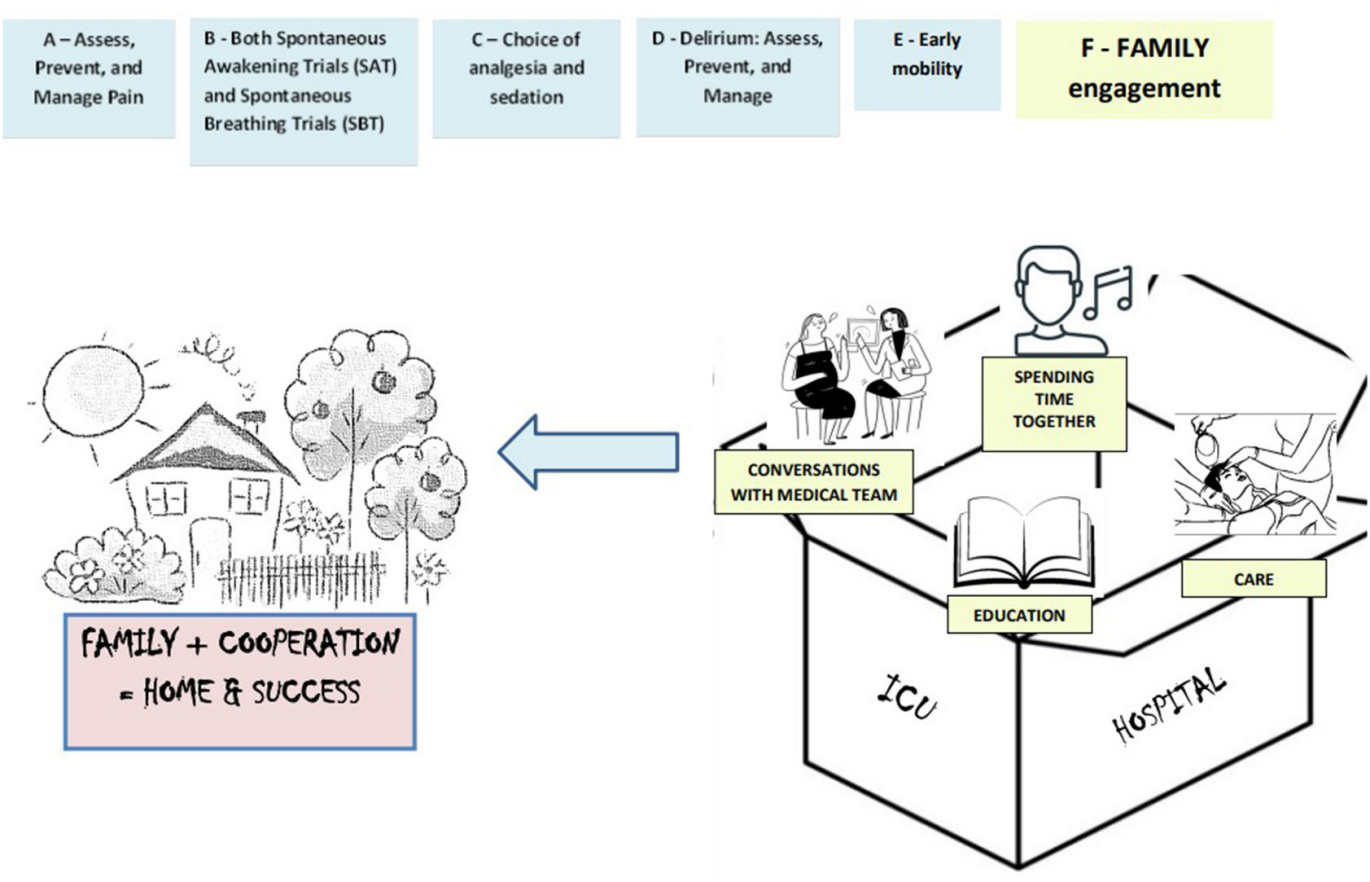
ABCDEF package.

Patients' families have an important role in ICU patient care. They are often involved in the decision-making process as representatives of critically ill patients, support their relatives and are the link between the patient and the ICU medical staff. Patient Centered Care (PCC) and Family Centered Care (FCC) is increasingly being implemented and desired in the hospital setting. The PCC and FCC model of care involves patient care that takes into account and respects the patient's beliefs, values and preferences, and involves the family in the process of caring for the relative (1, 2). Family involvement in the patient care process (F) in the intensive care unit has been added to the ABCDEF package. The ABCDEF package is an evidence-based approach to the holistic management of critically ill patients, with the focus on optimizing recovery and patient outcomes in the ICU and engaging and empowering patients and families during hospitalization. It includes: (1)**A**ssess, Prevent, and Manage Pain, (2) **B**oth Spontaneous Awakening Trials (SAT) and Spontaneous Breathing Trials (SBT), **C**hoice of analgesia and sedation, (3) **D**elirium: Assess, Prevent, and Manage, (4) **E**arly mobility and Exercise, and (5) **F**amily engagement and empowerment (Figure 1) (3, 4). Studies showed that greater compliance with the ABCDEF package was independently associated with improved clinical outcomes (5). ICU patients are at particular risk of developing delirium during hospitalization. It is estimated to occur in up to 80% of ICU patients (6). Studies show that implementing non-pharmacological interventions to prevent delirium, including interventions with the family, can reduce the incidence of delirium (7–9). Understanding the experiences and attitudes of carers can contribute to the development of nursing interventions with patient families, provide support, education and improve the relationship between medical staff and patient families. In addition, highlighting the role that ICU nurses play in their relationships with families/relatives of delirious ICU patients may result in increased staff awareness, which may ultimately have a positive impact on the care of delirious ICU patients and improve the wellbeing of patients and their families.

### Objectives and rationale

The aim of the scoping review was to search the available literature about the experiences and attitudes of family/carers of ICU patients diagnosed with delirium during hospitalization. Of particular interest were the experiences of relatives because these people are increasingly involved in the care of critically ill patients and the risk of delirium is high in ICU patients. The research questions (RQs) for our scoping review are as follows:

1. What is the family's experience of caring for ICU patients with delirium?

2. What is the attitude of the family toward the care of an ICU patient with delirium?

## Methods

### Study design

We chose the scoping review method because we wanted to map terms relevant to the involvement of relatives in the care of critically ill patients with delirium. Scoping reviews are a relatively new approach to synthesizing evidence, and there is currently little guidance on deciding between a systematic review and a scoping approach during the synthesis of evidence, especially when the literature has not yet been comprehensively reviewed or shows a large, complex or heterogeneous nature that cannot be subject to a more thorough systematic review (10).

We conducted the scoping review according to the methods described in the Joanna Briggs Institute Methodology Manual for Scoping Reviews (11), and using the recommendations of the Preferred Reporting Items for Systematic Reviews and Meta-analysis for Scoping Reviews (PRISMA-ScR) guidelines (12) (available on request from the corresponding author).

### Inclusion criteria

To identify important aspects related to family experiences and attitudes toward care of ICU patients with delirium, we developed research questions that clearly define the Population, Concept and Context (PCC) of the scoping review.

#### Population

Studies whose participants were family or carers of patients admitted to the intensive care unit who were diagnosed with delirium during the hospital stay were included in the review.

In this scoping review, adults were defined as those who were aged 18 years or older.

Family was defined as people who are related to patients by blood or marriage, and carers/relatives were defined as people who accompanied patients during their stay in the ICU.

#### Concept

The object of interest was the experiences and attitudes of family members/carers whose relatives experienced delirium while in the ICU stay. This included research on the experiences and readiness of relatives to be involved in the care of an ICU patient with delirium. In addition, we also included studies on the impact of caring for an ICU patient with delirium on the occurrence of anxiety and depression symptoms.

#### Context

Studies to be included in the review had to be conducted in the adult intensive care unit.

#### Types of studies

Quantitative and qualitative studies of any design or methodology were included in this review. Secondary evidence sources—literature reviews and systematic reviews—were also included.

### Exclusion criteria

Studies that took place in non-ICUs or children's ICUs were excluded from the scoping review. Studies in which the concept did not involve delirium and the experiences and attitudes of the family toward the care of an ICU patient with delirium were also rejected. In addition, studies published before 2017 and in a language other than English were excluded from the scoping review.

### Search strategy

Two authors systematically searched the following databases: PubMed, Scopus, EBSCO, Web of Science, and Cochrane Library. The following keywords were used: “care givers”, “family”, “experience”, “attitudes”, “delirium”, “delirium prevention”, “ICU”,” critical ill”. Keywords with their combinations using AND or OR were entered. The search was limited to studies published between 2017 and 2022. All publications were examined by title and abstract to exclude irrelevant records. Any discrepancies were resolved through discussion with the researchers, and at the end of the selection process, full agreement was reached on the articles to be included. The database search was ongoing from 15 July 2022, with a final search on 4 August 2022.

### Extraction of data

The data extraction form, based on the JBI scoping review guidelines (11), was used, and the most important information in the studies was included. Data extraction, which is referred to in the scoping review as “data charting” (13) was undertaken by two reviewers independently. To identify relevant studies, we used the Population-Concept-Context (PCC) framework. Information extracted from included studies included: First author, and year, country, study design, aim of the study, inclusion (PCC) and exclusion criteria, results, and findings. Reviews are considered eligible if all the following criteria are met. The authors performed the extraction using Microsoft Excel.

### Critical appraisal process

The purpose of this scoping review was to collate the information that has been published on family experiences and attitudes toward care of ICU patients with delirium. We did not critically appraise the individual sources of evidence. For a scoping review, it is acceptable to review the current evidence without considering the methodological assessment of the included studies (13).

## Results

**Figure 2:**
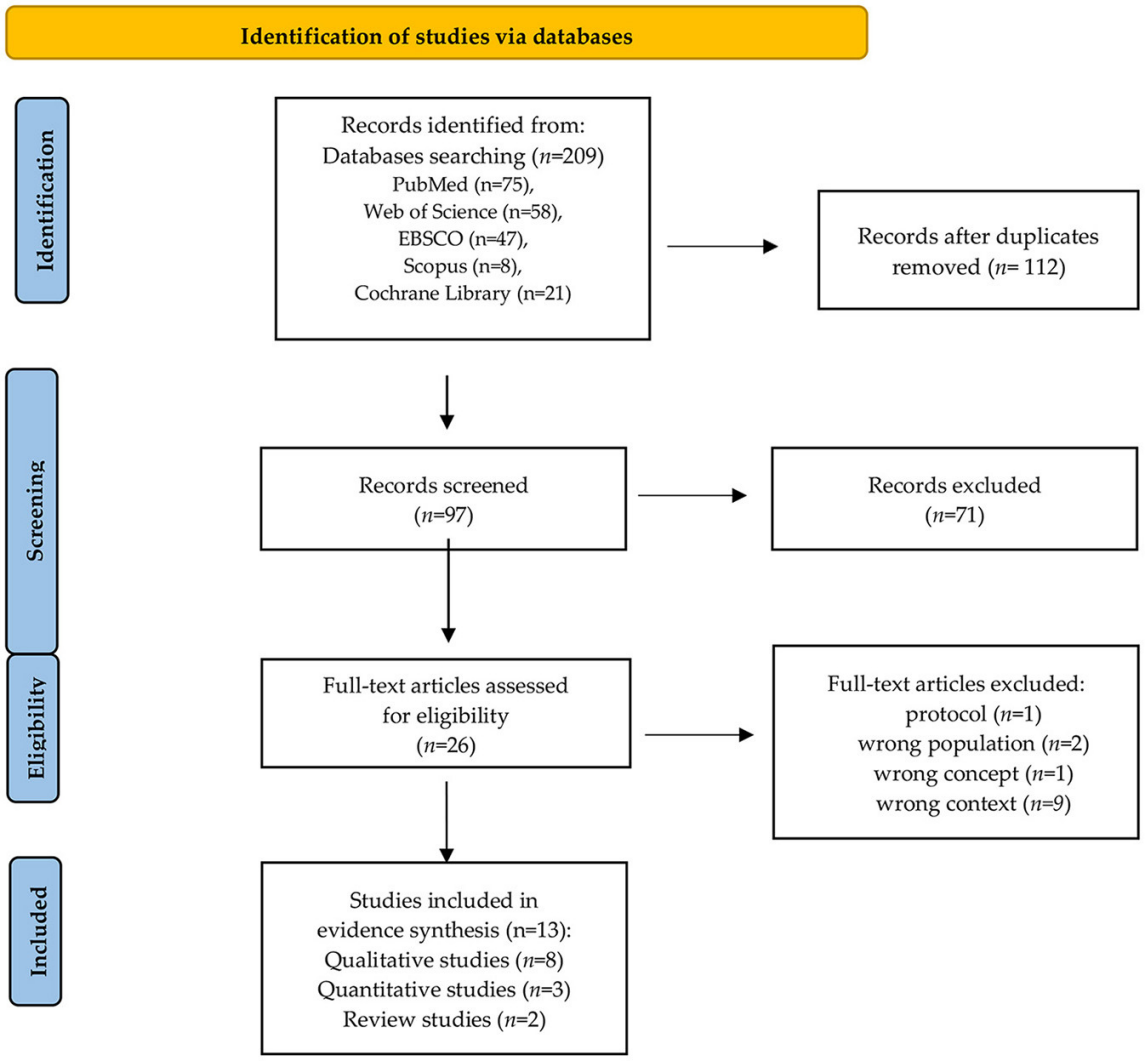
PRISMA flow diagram (14).

Our scoping review identified 209 articles, from which 13 articles reporting the experiences and attitudes of family/carers of ICU patients who developed delirium during hospitalization were included (Figure 2). Of the included studies, eight were qualitative studies (15–22), three were quantitative studies (23–25), and two were reviews (one systematic review and one integrative review) (26, 27). The studies were conducted in North America (*n* = 5), Europe (*n* = 4), South Africa (*n* = 1), Asia (*n* = 1). To reach the purpose, qualitative studies mostly used the semi-structured interview method. In quantitative research, questionnaires were used: Sour Seven, Patient Health Questionnaire 9 (PHQ-9), Generalized Anxiety Disorder 7 (GAD-7) and original survey questionnaires. The results and conclusions are presented in Tables 1–3.

**Table 1:** Characteristics and findings of qualitative study included in scoping review.

**References**	**Country**	**Study design**	**Participants**	**Findings**
Lange et al. (15)	Poland	Qualitative study (semi-structure interviews)	8 family members	✓ Inadequate education and information, ✓ The benefit of obtaining prior information, ✓ Surprise, shock, and anger at the change in a relative's behavior.
Leigh et al. (16)	Canada	Qualitative descriptive study (focus groups)	3 family members	✓ Family detection of delirium is feasible and of value for patient care and coping by family members. ✓ Actively involving family members in delirium detection at the bedside may improve outcomes and experiences for both patients and family members.
Huang et al. (17)	Taiwan	Qualitative descriptive study (semi-structure interviews)	20 family caregivers	✓ Uncertainty among family caregivers of patients with delirium in ICUs can lead to feelings of fear and anxiety.
Pandhal et al. (18)	England	Qualitative descriptive study (semi-structure interviews)	9 relatives	✓ Lack of understanding about delirium amongst family members and how they could have supported delirium management. ✓ Families were keen to be involved in delirium. ✓ Interventions such as video-ICU delirium education have been found to be effective in educating family members about delirium management.
Hume (19)	South Africa	Qualitative study (semi-structured interviews, unstructured observation and and focus groups)	2 family members	✓ The negative impact of the physical environment and pacing culture in intensive care. ✓ Damaging mistrust
Bohart et al. (20)	Danish	Qualitative study (semi-structure interviews)	11 relatives	✓ Lack of knowledge about delirium, ✓ Delirium as a second order problem, ✓ Lack of information on delirium by medical staff. ✓ Varied nature of delirium and different ways of dealing with it
Page et al. (21)	UK	Qualitative study (in-depth interviews)	15 family members	✓ Relatives experience the events associated with ICU hospitalization (including those associated with delirium) in a very real and ingraining manner. ✓ Family members may experience a different version of a critical illness episode than their relatives (patients).
Smithburger et al. (22)	United States	Qualitative study (purposeful sampling, interview)	10 family members	✓ Families of ICU patients want to be involved in the care and prevention of delirium. ✓ Need for communication between family and nursing staff

**Table 2:** Characteristics and findings of quantitative study included in scoping review.

**References**	**Country**	**Study design**	**Participants**	**Findings**
Poulin et al. (23)	Canada	A cross-sectional study	147 family caregivers	Family caregivers of critically ill adults with delirium frequently experience clinically significant anxiety and are significantly more likely to report frequently worrying too much about different things.
Smithburger et al. (24)	United States	A cross-sectional study	60 family members	Patients' families can be a valuable resource to engage when implementing delirium-prevention activities in the ICU, according to health care providers and family members
Rosgen et al. (25)	Canada	A cross-sectional study	147 patient-caregivers	Caregiver-detected patient delirium was associated with increased depression and anxiety symptoms in family caregivers of critically ill patients.

**Table 3:** Characteristics and findings of review study included in scoping review.

**References**	**Study design**	**Included studies**	**Participants**	**Findings**
Boehm et al. (26)	A systematic review and qualitative meta synthesis	14 articles	364 participants inc. 70 family members	✓ The experience of delirium has emotional, cognitive, physical, relational, spiritual, and situational implications for patients and family members. ✓ Less attention is focused on the interventions that patients and their families perceive as beneficial in alleviating this suffering.
Assa et al. (27)	An integrative review	7 studies	633 family caregivers	✓ Family caregivers experienced adverse outcomes related to delirium in patients in the ICU (e.g., distress, anxiety, depression, anger, shock, uncertainty, dissatisfaction). ✓ Family Caregivers' needs: informational support, emotional support from health care professionals effective communication.

## Discussion

In this review, we identified 13 studies regarding the experiences and attitudes of families/carers of ICU patients who experienced delirium during hospitalization, published between 2017 and 2022. Our findings show that carers experienced adverse effects associated with delirium in ICU patients such as stress, anxiety, embarrassment, uncertainty, anger, shock. Families/ relatives need both emotional and informational support from medical staff. Relatives want to be involved in the care of a patient with delirium, but this requires removing some of the barriers such as: lack of family knowledge and awareness of delirium, improved education, and communication with medical staff. Research shows that the use of delirium assessment tools designed for the family is feasible and acceptable to medical staff. Family involvement may induce an increased anxiety but this aspect needs further research. The included studies support family involvement in the care of ICU patients with delirium.

Research shows that the family ('F') is important in the care of the ICU patient, therefore experts have included the family in the ABCDE package developed (5). Critical illness affects not only the patient himself, but also his family members and other caregivers (e.g., partners, friends) who participate in the care process of the ICU patient. Therefore, learning about the experiences and attitudes of these people is fundamental to recognizing their needs and involving them in the care process (3). The PCC and FCC model of care, which involves taking into account the wishes, needs, questions, concerns of patients and their families, is increasingly desirable in the care of critically ill patients (1). Family members/carers often become surrogates and participate in decision-making regarding ICU patients. As family/carers/relatives are the people who know the patient best they are able to indicate the patient's likes and preferences, but can also more easily identify changes in the patient's behavior that may be indicative of delirium symptoms (16). It is therefore important that the family's opinion is heard and taken into account in the decision-making process (28). The positive impact of family involvement has been shown even during traumatic events such as cardiopulmonary resuscitation (CPR). In a study by Jabre et al. (29), it was shown that family presence during CPR was associated with positive outcomes on psychological variables, did not interfere with medical efforts and did not increase stress in the medical team or cause medico-legal conflicts.

A study by Leigh et al. (16) showed that medical staff, patients, and carers think it is feasible for family members to detect delirium. The family, who know the patient, their behavior and manner, can provide valuable information about subtle changes in the patient's behavior. Which can positively influence patient care. Similarly, in a study by Smithburger et al. (24) most medical staff (93% of doctors and all nurses) believed that the family could be involved in delirium prevention. Family involvement will result in increased time spent on delirium prevention activities, which may result in a reduced incidence of delirium in the ICU.

Our findings suggest that family/relatives of ICU patients want to be involved in delirium-related activities (16, 18, 22). In addition, family involvement in care by performing tasks such as delirium assessment, non-pharmacological interventions to prevent delirium, provide family members with a sense of purpose and may be a protective mechanism to reduce their stress related to their relative's critical illness (18). Similarly, in the Smithburger et al. (22) study, family/relatives were willing to engage in non-pharmacological interventions to prevent delirium. Moreover, the family felt that their presence and interventions such as: reading newspapers, news, providing items that patients used on a daily basis, e.g., electric shaver, calendar, and boards to help them communicate, had a positive impact on the patient, particularly during an episode of delirium.

Both medical staff, patients and family are of the opinion that relatives can and should be involved in delirium prevention activities, but there are several barriers that should be considered (16, 18, 24). The most important of these is the low level of knowledge and awareness of delirium among family members (15, 16, 18, 20). The results of a study by Bohart et al. (20) showed that delirium is an unknown term to relatives, there is little knowledge about it and delirium symptoms are taken as a natural consequence of critical illness and are not a primary concern in ICU care. Similarly, in a study by Lange et al. (15) families assigned delirium symptoms as a consequence of anesthesia. In the Leigh et al. (16) study, focus group participants (including family) unanimously reported that families were likely to have moderately low to low levels of knowledge about delirium. Patients and their families confessed that they learned about delirium by searching the Internet or reading brochures given to them by the research team. In the Pandhal (18) study, participants said that a lack of knowledge about delirium and critical illness made it difficult to meet a relative staying in the ICU. At the same time, they highlighted the fact that understanding delirium would facilitate the patient's mental health recovery. Which was also expressed by family and patients in the study by Lange et al. (15).

According to families and relatives, one of the barriers was inadequate communication between medical staff and carers (15–19, 22). Families often feel uncertain about the interventions they are allowed to perform with their relative. This is due to the lack of clear communication from bedside nurses. Increasing the comfort of families would be influenced by an invitation from nurses to participate in care (22). Another study by Smithburger et al. (24) found that only 28% of participants were discussed with medical staff about participating in delirium prevention activities. Similarly, in a study by Huang et al. (17), in which difficulties in decision-making were due to caregivers' lack of knowledge about the patient's medical needs, as well as limited time to communicate with staff. The opportunity to talk and get information from medical staff about delirium brings relief to the family (15, 20). In a study by Bohart et al. (20) participants who received information from staff about delirium described it as a relief. This helped them to understand the changes in their relative's behaviors. This was also noted by participants in the Lange et al. (15) study. In a study by Smithburger et al. (24) participants found talking to a bedside nurse about the patient's concerns to be the best strategy for reducing the discomfort of involvement in care. In a qualitative meta-analysis by Boehm et al. (26) it was shown that patients and family members valued simple, empathic interpersonal communication and clinician interventions related to delirium as a means of relieving distress.

An important facilitating factor for family involvement, according to families and relatives, is education. Families feel the need and want to be educated about delirium (15–18, 20). The best way to educate according to families in the Huang et al. (17) study, was a one-on-one conversation with a medical professional. Similarly, in the Smithburger et al. (22) study, 55% of participants indicated that one-on-one education with a healthcare professional was the best approach. Video education was found to be an effective education intervention. This allowed family members to absorb information at their own speed while avoiding the workload of staff (18). Indirect education, using written information about delirium, brochures, is also an acceptable form (20). However, families emphasize the need for dialogue with medical staff. This will enable a thorough understanding of the clinical context of delirium (16, 20).

Families experience negative emotions related to the occurrence of delirium in their relatives such as: stress, anxiety, embarrassment, uncertainty, anger, shock (15, 27). Consequently, their needs are not only focused on providing informational but also emotional support. The results of studies on the impact and detection of delirium on the occurrence of symptoms of stress, anxiety and depression in relatives of patients are unclear (25). On the one hand, the outcome of delirium detected by the caregiver was related to the severity of anxiety symptoms such as: “Feeling nervous, anxious or tense”; “Inability to stop or control worry” (23). In the Rosgen et al. (25) study, 26.5% of family carers reported clinical symptoms of depression and, 35.4% reported clinical symptoms of anxiety. The results of the study showed a significant but variable association between delirium detected by families and symptoms of depression and anxiety. In contrast, other studies suggest that family assistance and involvement in ICU care tasks can give family members a sense of purpose, be supportive and serve as a protective mechanism to reduce stress (30). In a study by Smithburger et al. (24) the family's comfort level with non-pharmacological interventions to prevent delirium such as light management, use of earplugs, eye curtains, cognitive stimulation, reorientation, playing music, providing glasses, hearing aids were assessed. The results showed an overall median for all activities of 9, where 10 represented total comfort. In addition, participants in this study did not consider barriers such as fear of having an intravenous catheter or tube removed to be a source of stress for them. Among the best ways to reduce the discomfort of participating in care, 68% of families/relatives reported talking with the nurse. This highlights the need for increased awareness among nursing staff about delirium and their role as educators for the patient and their families.

A high incidence of delirium is also observed in non-ICU wards (31). Many of the non-pharmacological interventions with the family used in ICUs can also be successfully implemented in other hospital wards. Providing patients with daily living equipment (glasses, hearing aids), equipment to improve sleep quality (earplugs, blindfolds), and stimulating cognitive function by providing current newspapers, radio, talking to relatives about family events can be interventions in which patients' families will be involved.

## Limitations

Some important studies may have been omitted from the search and selection process due to limitations on publication date (studies from 2017) and language (English). The studies included in the review were conducted in different regions, so the cultural aspect and the general policies of the ICU environment in the country should be considered.

## Conclusion

Family/carers of ICU patients are the people who can provide information about the initial symptoms of delirium in ICU patients. Relatives want to be involved in the care of a patient with delirium, but this needs improvement in some aspects of care such as: lack of awareness, family/ relatives' knowledge of delirium, improved education, and communication with medical staff. Recognition of delirium by families is acceptable and feasible. Involvement of the family may result in an increased anxiety, but this aspect needs further research.

## Implications for research

Further research on the experiences of families and ICU patients related to delirium are needed. Understanding the perspective and experiences of patients and their families related to an episode of delirium is an important part of the management of delirium. Conducting additional interviews could potentially reveal added information, perspectives, experiences of delirium. Additionally, future research should consider the psychological aspect of the impact of family involvement in the care and detection of delirium in their relatives.

## Implications for practice

Difficulties in caring for patients with delirium often result from a lack of knowledge of delirium by family/relatives. It is crucial to provide education in this area. Educating the family/relatives with ready-made brochures or videos is effective but should be complemented by a direct conversation with medical staff, which is the method most preferred by families. Education should be conducted at the beginning of the patient's stay in the ICU. In addition to theoretical education, families need emotional support from the medical staff (e.g., by talking to the nurse at the patient's bedside), which bring them relief, alleviate anxiety, and reduce discomfort. Communication between medical staff and the family should be based on clear communication of expectations from both sides and have an open dialogue. There is a concern that family involvement in detecting symptoms of delirium can be a stressor, so these people should be offered support from a professional such as a psychologist.

## Author contributions

Conceptualization and methodology: SL, WM-D, and SK. Formal analysis: SL and SK. Writing—original draft preparation: SL, WM-D, AF, and SK. Writing—review and editing: SL, WM-D, AF, DR, and SK. Supervision: DR and SK. All authors have read and agreed to the published version of the manuscript.

## Conflict of interest

The authors declare that the research was conducted in the absence of any commercial or financial relationships that could be construed as a potential conflict of interest.

## Publisher's note

All claims expressed in this article are solely those of the authors and do not necessarily represent those of their affiliated organizations, or those of the publisher, the editors and the reviewers. Any product that may be evaluated in this article, or claim that may be made by its manufacturer, is not guaranteed or endorsed by the publisher.
